# Changes in Arsenic Speciation in Wild Edible Fungi after Different Cooking Processes and Gastrointestinal Digestion

**DOI:** 10.3390/molecules28020603

**Published:** 2023-01-06

**Authors:** Yang Liu, Shaozhan Chen, Qianyu Li, Liping Liu

**Affiliations:** 1 Beijing Key Laboratory of Diagnostic and Traceability Technologies for Food Poisoning, Beijing Center for Disease Prevention and Control, Beijing 100013, China; 2 School of Public Health, Capital Medical University, Beijing 100069, China

**Keywords:** arsenic, As speciation, bioaccessibility, edible fungi

## Abstract

Arsenic (As) is enriched in wild edible fungi, which is one of the main important sources of As in humans’ diet. In this study, two wild edible fungi were employed for investigation: (1) *Pleurotus citrinopileatusone*, which contains a high content of inorganic As (iAs) and (2) *Agaricus blazei Murill*, which contains a high content of organic As. This study investigated the changes in As content and its speciation after different daily cooking methods. We found that the content of As in *Pleurotus citrinipileatus* and *Agaricus blazei Murill* reduced by soaking plus stir-frying by 55.4% and 72.9%, respectively. The As content in *Pleurotus citrinipileatus* and *Agaricus blazei Murill* decreased by 79.4% and 93.4%, respectively, after soaking plus boiling. The content of As speciation in dried wild edible fungi reduced significantly after different treatments. Among them, iAs decreased by 31.9~88.3%, and organic As decreased by 33.3~95.3%. This study also investigated the bioaccessibility of As in edible fungi after different cooking processes via an in-vitro physiologically based extraction test (*PBET*). The results showed that the bioaccessibility of As was relatively high if the edible fungi were uncooked, boiled, or stir-fried. The gastric (G) bioaccessibility of As ranged from 51.7% to 93.0% and the gastrointestinal (GI) bioaccessibility of As ranged from 63.5% to 98.1%. Meanwhile, the bioaccessibility of inorganic As was found to be as high as 94.6% to 151%, which indicates that further evaluation of the potential health risks of wild edible fungi is necessary.

## 1. Introduction

Wild edible fungi are considered to be a type of food with a relatively high nutritional value. In recent years, the worldwide consumption of edible fungi has increased dramatically. They are rich in protein, essential amino acids, and other nutrients [1,2]. In addition, edible fungi possess a high potential for use as medicines for disease treatment due to their medicinal properties. Edible fungi have hundreds of different medicinal functions, such as being anti-allergy, anti-diabetes, anti-cancer, etc. [3,4].

Aside from their nutritional value, edible fungi tend to accumulate toxic chemical elements such as As, cadmium, mercury, and lead in soil and water [5,6]. Among these elements, As is more inclined to be enriched and accumulated via biotransformation. The content and speciation of As vary in different edible fungi [7,8,9]. The toxicity of As mainly depends on its speciation. iAs is much more toxic than organic As. Arsenic Choline (AsC) and Arsenic Betaine (AsB) are considered to be nearly nontoxic [10,11]. Therefore, it is necessary to study the As speciation of edible fungi.

Most people are exposed to As through contaminated water or food [12]. Long-term exposure may pose a threat to human health. In this case, it is necessary to conduct a risk assessment of the potential impacts on consumers. Food consumption-related risks could be quantified by evaluating the dose of toxic contaminants that consumers are exposed to. The dose depends not only on the total content of toxic contaminants in the food but also on the bioaccessibility of contaminants in the food matrix [13].

Bioaccessibility is defined as the proportion of a substance that is absorbed through the intestinal epithelium, enters an organ or bloodstream, and reaches the body’s circulation to exert its effect on the gastrointestinal tract. The bioaccessibility of As can be studied either in vivo or in vitro. In vivo experimental studies are more complex, involving ethics-related issues. Existing studies indicate a good correlation between the results from in vitro studies and those from in vivo studies [14]. Currently, the in vitro model used for in vitro studies is mostly *PBET* which mimics the extracts from gastric and small intestinal digestion stages [15]. This approach can approximate the in vivo condition effectively, while the experimental conditions for *PBET* are easier to control than for in vivo studies [16]. Additionally, *PBET* offers many advantages, including simplicity, convenience, and low cost, etc.

As a rule, edible fungi are generally consumed after being cooked which adds to the flavor. Studies have shown that different cooking methods can alter the chemical speciation and content of As in food, accordingly affecting the bioaccessibility of As [17]. Therefore, it is necessary to consider the impact of cooking methods while assessing the risks of edible fungi. In a recent study [18], the As content and bioaccessibility of As in three types of edible fungi—*Aagaricus bisporus*, *Shiitake*, and *Pinnacle*-before and after cooking were measured by the in vitro *PBET* method. However, this study only analyzed total As. Total As cannot provide accurate information on the potential health risks associated with edible fungi, because the toxicity of different As speciation is different [19].

So far, most related studies were conducted using fresh fungi. The available literature on dried edible fungi is limited, since most of the wild edible fungi cannot be artificially cultivated under normal conditions, and their seasonality is another problem. Currently, water removal followed by dry storage is the most common way to preserve wild edible fungi. Dried edible fungi are easy to preserve, their consumption has increased in recent years [20]. As earlier studies [21] have shown, dried fungi not only does not affect their nutritional value but also prolongs their storage time. Chiocchetti et al. [22] characterized the bioaccessibility of Hg, Cd, As, and Pb in dried edible fungi. They found that the bioaccessibility of As was higher than that of other metals. However, this study only involved the bioaccessibility of total As, not considering the bioaccessibility of diverse As speciation in dried edible fungi. Thus, our study focused on As and its speciation changes in wild edible fungi under different cooking methods and in bioaccessible extracts, which can more accurately express the health risk of arsenic in wild edible fungi to humans.

## 2. Results and Discussion

### 2.1. As Speciation Analysis Method

**Figure 1:**
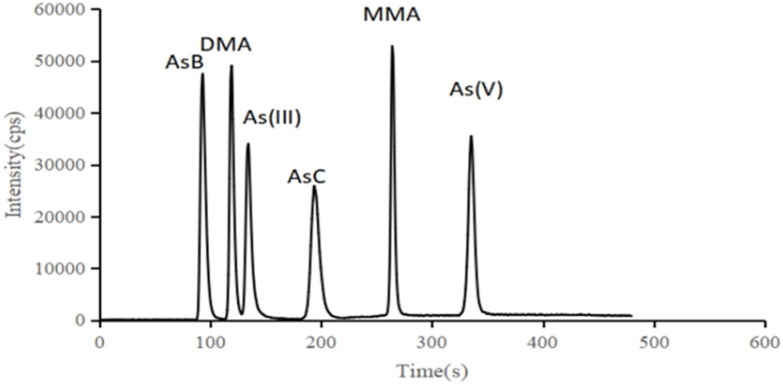
Standard chromatogram (10 μg L^−1^).

The As speciation in the raw and cooked edible fungi, in vitro bioaccessible fractions were separated by HPLC-ICP-MS. The established method can complete the separation of six species (AsB, DMA, As(III), MMA, AsC, As(V)) in 8 min (10 μg L^−1^ standard chromatogram is shown in Figure 1). The results indicate that the linearity ranges for the six As species were all between 0 and 100.0 μg L^−1^ with linear correlation coefficients more than 0.999. The detection limits were between 0.05 μg L^−1^ and 0.15 μg L^−1^. The reproducibility of this method was evaluated by precision under different concentrations. The relative standard deviations (RSD) of the six species were less than 5.0% and the recoveries of all concentration levels ranged from 80% to 120%. This method is sensitive, quick, reproducible and accurate, making it suitable for the rapid determination of As speciation in edible fungi.

### 2.2. Quality Assurance and Quality Control

To assess the accuracy of the method for evaluating total As, two CRMs (ERM-BC211, NIST SRM 1568b) were included in the digesting and analyzing procedures for quality assurance and quality control. The measurement results are shown in Table 1. The measured values of both standards are within the standard values and the RSD are less than 5%, which shows that the determination method of total arsenic is accurate and reliable. 

### 2.3. As Content in Soaked and Cooked Edible Fungi

#### 2.3.1. Total As Content and Its Speciation in Soaked Edible Fungi

In this study, wild dried edible fungi were used as the research object. According to people’s daily cooking habits, the cooking is usually done after soaking. Therefore, the soaked edible fungi were used for subsequent cooking in this study. The total As content and As speciation of the edible fungi before and after cooking are shown in Table 2 (all the results in the study are expressed as dry mass). After soaking, approximately 35.2% and 60.9% of As from *Pleurotus citrinipileatus* and *Agaricus blazei Murill* was dissolved in water, respectively. The As speciation in the soaking solution were also measured. Overall, 37.1% of iAs was released from the iAs-dominated fungus, *Pleurotus citrinipileatus,* during the soaking procedure. DMA, MMA and AsB were released by 83.0%, 90.8%, and 62.8%, respectively. Overall, 61.9% of AsB was released from organic As-dominated fungus, *Agaricus blazei Murill,* during soaking, while DMA, MMA and iAs were released by 66.7%, 47.7% and 77.7%, respectively. The results indicate that soaking removed some As speciation of the edible fungi, and the released As speciation during the soaking procedure was the same as the initial As speciation in the food matrix. Hanaoka et al. [23] and Laparra et al. [24] found the concentration of total As in hijiki is lowered by as much as 60% in the process of washing and soaking. However, this study did not determine the As speciation in the soaking solution.

#### 2.3.2. Total As Content and Its Speciation in Cooked Edible Fungi

**Figure 2:**
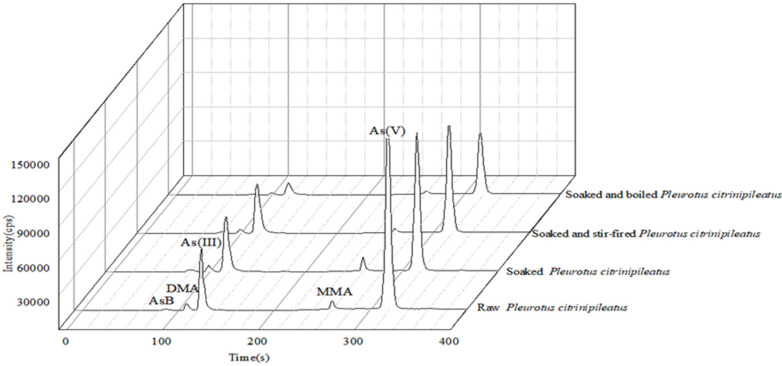
As speciation changes in *Pleurotus citrinipileatus* after different cooking methods.

**Figure 3:**
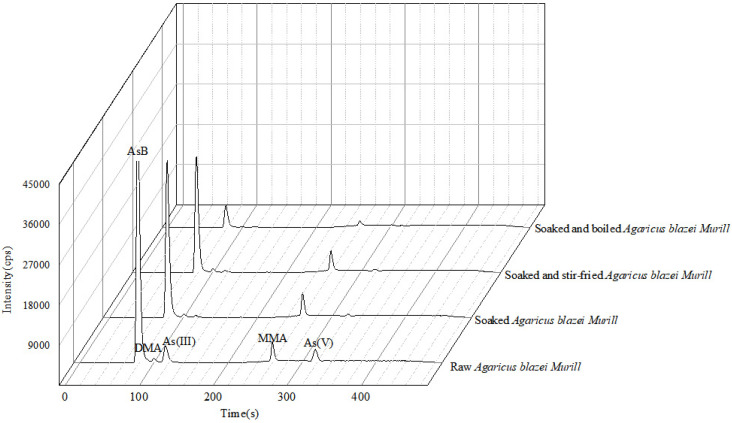
As speciation changes in *Agaricus blazei Murill* after different cooking methods.

The As content of the edible fungi decreased to some extent after soaking, boiling, stir-frying. Both total As and its speciation in cooked edible fungi were lower than those in raw edible fungi (raw > soaking plus stirfrying > soaking plus boiling). Compared with the raw edible fungi, soaking plus boiling reduced 79.4% and 81.4% of the total As content and iAs in *Pleurotus citrinipileatus*. The As content in *Agaricus blazei Murill* was reduced by 93.4%, including the 95.8% decrease in AsB. The changes in As speciation and total As after different cooking processes are shown in Table 2. The chromatogram of changes in As speciation after different treatments of *Pleurotus citrinipileatus* and *Agaricus blazei Murill* are shown in Figure 2 and Figure 3. The results indicate that the proportion of As leaching was higher after soaking and boiling. Dried edible fungi are often used for cooking soups. Boiling increases the contact movement between water and fungi, which enhances the release of As. Compared to soaking only (51.5–59.3%reduction), more As was reduced after soaking plus boiling (79.4–93.4% reduction) in the two dried edible fungi. Cheyns et al. [25] discovered that As in hijiki was reduced by about 50% after boiling compared to soaking only (28% reduction) and As in boiled rice and vegetables was reduced by 53–66% and 12–43%, respectively. In contrast, Liu et al. reported the As concentration of rice after soaking and cooking did not change significantly from that before treatment [26]. The contrary consequence may be due to the difference between food matrices. Therefore, soaking and boiling raw edible fungi could reduce most of the iAs intake and reduce the risk of consumption. The As content in the soup of boiled edible fungi was high, which suggested that drinking the soup while boiling the edible fungi should be avoided. Soaking plus stirfrying resulted in significant differences (*p* < 0.05) in the As content of *Pleurotus citrinipileatus* and *Agaricus blazei Murill* before and after cooking. The As content was reduced by about 55.4% and 72.9%, respectively. Toni et al. [18] studied the effect of different cooking methods on the As content in three different edible mushrooms, *Shiitake*, *Agaricus bisporus* and *P. ostreatus*. The total As levels of these three mushrooms are 1393, 181 and 335 ug kg^−1^, respectively. They reduced by 53% to 71% after boiling and by less than 11% after roasting. However, we could not compare the changes in As speciation in these edible fungi because the study did not include such an investigation.

The As speciation (AsB, DMA, MMA, iAs) studied decreased between 80.2% and 84.9% in *Pleurotus citrinipileatus* after soaking plus boiling, which resulted in a decrease of about 15.4% to 55.6% after soaking plus stir-frying Among them, iAs was reduced by 81.4% and 55.6%, respectively. after being treated with two cooking methods, and the decrease was more obvious with the soaking plus boiling method. Each As speciation in *Agaricus blazei Murill* was treated by soaking plus boiling and soaking plus stir-frying methods to decrease about 86.7–95.8% and 36.2–72.4%, respectively. Furthermore, among them, iAs decreased by 88.3% and 36.2% respectively. The effect of the two methods on As speciation was different (*p* < 0.05), and it can be seen that the use of soaking plus boiling resulted in a significant decrease in the content of the individual As speciation of both edible fungi.

### 2.4. Bioaccessible As in Raw, Soaked and Cooked Edible Fungi

#### 2.4.1. Bioaccessible As in Raw and Soaked Edible Fungi

The bioaccessibility of As content in raw and soaked edible fungi during the in vitro G and GI fractions are shown in Table 3. The results clearly indicate the differences in the bioaccessibility of As in these two wild edible fungi (*p* < 0.05). The G and GI fractions of raw *Pleurotus citrinipileatus* were 74.5% and 78.2%, respectively, while the G and GI fractions of raw *Agaricus blazei Murill* were 93.0% and 98.7%. This conclusion is similar to the results obtained in previous studies. Toni et al. [18] reported that the bioaccessibility of As in three mushrooms (*Shiitake*, *Agaricus bisporus* and *Pinnacle*) ranged from 86% to 97%. Koch et al. [13] found that the bioaccessibility of As for several wild mushrooms ranged from 22% to 94%. To be specific, *Laccaria laccata* had a bioaccessibility of As of 22%, and the bioaccessibility of As in *Suillus luteus* and *Agaricus* ranged from 50% to 60% and from 58% to 94%, respectively. Such evidence suggests the bioaccessibilty of As varied in different species of edible fungi. Similarly, He et al. [27] concluded that the bioaccessibility of As in different types of rice varied greatly.

The bioaccessibilities of iAs, AsB, DMA, and MMA in raw edible fungi (*Pleurotus citrinipileatus,* and *Agaricus blazei Murill*) were at a high level. To be specific, it ranged from 99.5% to 102.7%, from 99.2% to 99.3%, from 81.9% to 97.8%, and from 81.3% to 104.8%, respectively. Moreda-Piñeiro et al. [28] evaluated the bioaccessibility of total As and different species of As (AsB, DMA, As(III), AsC and As(V)) in seafood samples. The bioaccessibility of total As, AsB, DMA, As(III) and As(V) was higher than 83%, 71–106%, 95–114%, 87–106%, and 90–113%, respectively, which is consistent with the results of our study. It demonstrates that As and its speciation were highly bioaccessible in different food matrices.

#### 2.4.2. Bioaccessible As in Cooked Edible Fungi

**Figure 4:**
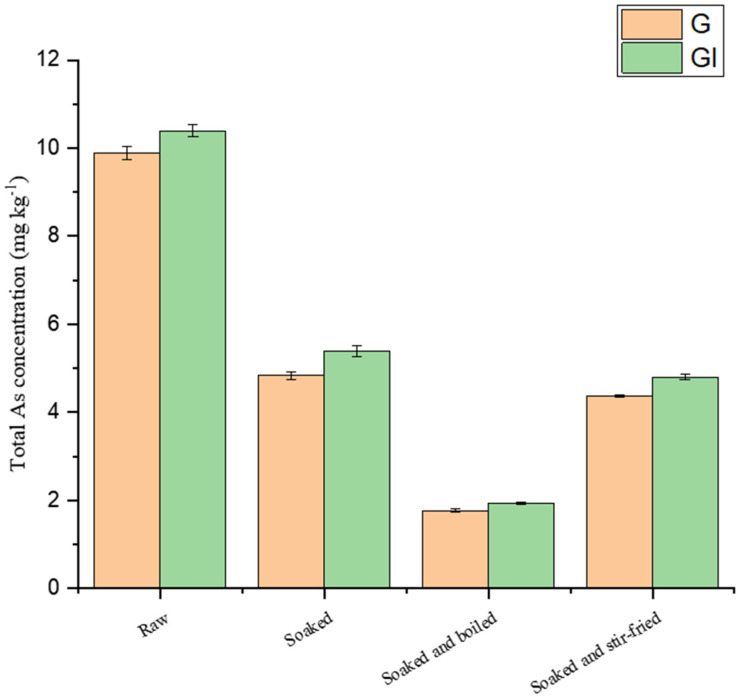
As content in G and GI fractions of *Pleurous citrinipileatus* after different treatments.

**Figure 5:**
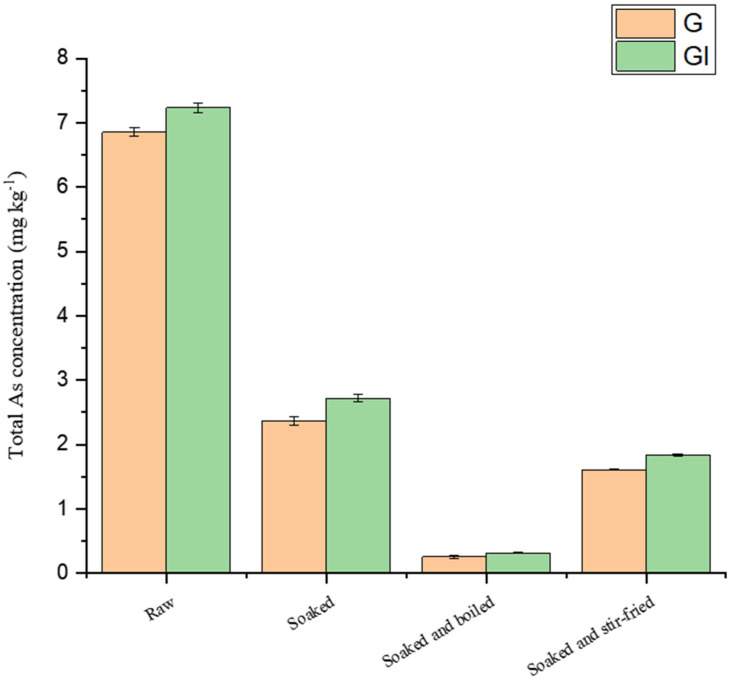
As content in G and GI fractions of *Agaricus blazei Murill* after different treatments.

Several studies on the bioaccessibility of As in rice and seafood have been reported [26,27,29]. However, a few studies on the bioaccessibility of As and its speciation in wild edible fungi have been conducted, especially the bioaccessibility of As in wild edible fungi under different cooking methods. Fu et al. [30] reported the bioaccessibility of As and its speciation in fish, shellfish and seaweeds after cooking. The bioaccessibility of total As and AsB differed significantly in different types of seafood. The bioaccessibility of As and its speciation in *Pleurotus citrinipileatus* and *Agaricus blazei Murill* under different cooking treatments are shown in Table 3. The variation in the bioaccessibility of total As in *Pleurotus citrinipileatus* and *Agaricus blazei Murill* are shown in Figure 4 and Figure 5. The bioaccessibility of As in edible fungi varies depending on the method of cooking. In this study, the bioaccessibility of As in *Pleurotus citrinipileatus* and *Agaricus blazei Murill* was 78.2% and 98.1%, respectively. The bioaccessibilities of As in *Pleurotus citrinipileatus* and *Agaricus blazei Murill* were reduced by soaking plus boiling from 78.2% to 63.5% and from 98.1% to 70.8%, respectively. Soaking plus stir-frying resulted in an increase in the bioaccessibility of As in *Pleurotus citrinipileatus* (from 78.2% to 81.0%) and a decrease in the bioaccessibility of As in *Agaricus blazei Murill* (from 98.1% to 92.0%). However, in a similar study, Toni et al. showed [18] that griddling and boiling improved the bioaccessibility of As in edible mushrooms, which may be related to the selected kinds of edible fungi. Hu et al. [31] studied the bioaccessibility of As in carrots, which ranged from 41.3% to 71.7% after steaming, boiling, and stir-frying, which was smaller than the bioaccessibility of As in carrots before cooking (77.2%). It validated that the bioaccessibilities of As in different food matrices were affected differently by diverse cooking methods.

The bioaccessibilities of different As species are presented in Table 3. Similar to the bioaccessibility of total As, the bioaccessibility of As speciation in the GI fraction was greater than that in the G fraction. This consequence may be attributed to the fact that As in food is digested by the gastric juice and subsequently by the intestinal juice. The bioaccessibility of AsB in the G fraction ranged from 84.3% to 95.6%, and in the GI fraction, it ranged from 95% to 105%, which was consistent with the bioaccessibility of AsB in seafood samples (65–100%) [32]. It was found that the bioaccessibilities of DMA and MMA in edible fungi ranged from 86.9% to 116.7% and from 81.3% to 114.5%, respectively, while the bioaccessibility of DMA and MMA in the shellfish ranged from 21.3% to 58.3% and from 3.4% to 59.2% [33]. It is hard to make a comparison across these two studies due to the different food matrices. Among the studied edible fungi, the bioaccessibility of iAs ranged from 91.5% to 151%. The high bioaccessibility may be due to the conversion of other As species into iAs during the *PBET* process. Furthermore, Leufroy et al. found the bioaccessibility of iAs in shellfish reached 188% [34]. They hypothesized DMA and MMA may be converted to iAs during *PBET*. To the best of our knowledge, this phenomenon has not be reported so far.

## 3. Materials and Methods

### 3.1. Instrumentation

An inductively coupled plasma mass spectrometry (ICP-MS) Agilent 7700X (Agilent Technologies, Darmstadt, Germany) with an autosampler (ASX-520) was fitted with an octupole/reaction cell. Helium was applied as the collision gas to reduce the argon chloride (^40^Ar^35^Cl) interference with As at *m*/*z* = 75. Chromatographic separations were carried out using the Agilent Technologies 1260 Infinity Series chromatography system (Agilent Technologies, Darmstadt, Germany). A Dionex IonPac AS7 anion exchange chromatographic column (4 × 250 mm) and its guard column Dionex IonPac AG7 (4 × 50 mm) (Dionex, Sunnyvale, CA, USA) were used for separation of the As speciation. A microwave digestion system (Preekem, Shanghai, China) was used for substance digestion. A digital control ultrasonic cleaner (KQ 500 DV, Kunshan, China) was used for the extraction of As speciation and in vitro *PBET*.

### 3.2. Chemicals and Standards

Standard solutions of AsB, DMA, AsC, MMA, As(III), and As(V) were all obtained from the National Institute of Metrology (Beijing, China). Working standard solutions (0 to 100 μg L^−1^) of As speciation were freshly prepared with 0.05% ammonia dilutions of corresponding stock solutions (1000 μg L^−1^). Nitric acid (69%, Merck, Darmstadt, Germany) was used in microwave-assisted acid digestion procedure. Ultrapure water (Milli-Q, Millipore, Billerica, MA, USA), Methanol (Sigma-Aldrich, Saint Louis, MO, USA), and ammonium carbonate (analytical reagent grade, Sinopharm Chemical Reagent Co., Ltd., Shanghai, China) were utilized to prepare the mobile phase. Hydrogen peroxide, pepsin, citric acid, maleic acid (99%), DL-lactic acid (Sigma-Aldrich, Saint Louis, MO, USA), hydrochloric acid and glacial acetic acid (100%, Merck, Darmstadt, Germany) were used for the gastric solution. Sodium hydrogen carbonate (Merck, Darmstadt, Germany), porcine bile salts, amylase and pancreatin (Sigma-Aldrich, Saint Louis, MO, USA) were used for the gastrointestinal solution. Three certified reference materials (CRMs) were analyzed for quality control in this study. ERM-BC211 and NIST SRM 1568b rice were obtained from the NIST (NIST, Gaithersburg, MD, USA).

### 3.3. Samples and Sample Pretreatment

In this work, 50 samples of wild edible mushrooms (dried wild edible fungi) were purchased on-line or from local markets, of which *Pleurotus citrinipileatus* and *Agaricus blazei Murill* with high As content were chosen for investigation. *Pleurotus citrinipileatus* with a high inorganic As content, purchased from the Yunnan Province of China, and *Agaricus blazei Murill* with high organic As content, purchased from the JiangXi Province of China were selected. We removed the inedible part of the dried samples. Based on daily dietary habit, 50 g from each sample were taken and soaked in 1000 mL of ultra-pure water for 30 min. After soaking, the soaked edible fungi were drained with a dry towel, cut into small pieces, mixed fully, and randomly separated into three groups of equal content. Each group was cooked under different processes. The soaked edible fungi and soaking solution were stored in a refrigerator at 4 °C.

### 3.4. Cooking Procedures for Edible Fungi

#### 3.4.1. Boiling

A total of 20 g soaked edible fungi was weighed and put into an ionized water-rinsed pot, to which 500 mL of deionized water were added. The edible fungi were boiled for about 10 min. The edible fungi soup was filtered and stored in 4 °C for subsequent experiments.

#### 3.4.2. Stir-Frying

A total of 20 g soaked edible fungi was weighed and put into an ionized water-rinsed pot, to which 1 g of edible oil was added. The fungi were stir-fried on low heat for about 10 min.

#### 3.4.3. Sample Homogenization

All treated edible fungi were chopped until completely homogenized by stainless steel chopping machine.

### 3.5. In Vitro PBET

The in vitro digestion method [14] was modified and applied to the edible fungi in this study. There were two consecutive extraction steps (gastric (G) and gastro + intestinal (GI)) for *PBET*. In the G step, 1 g of the raw or cooked edible fungi sample was placed in a 50 mL polypropylene centrifuge tube. 20 mL of freshly prepared gastric solution (1.25 g L^−1^ pepsin, 0.50 g L^−1^ citric acid, 0.50 g L^−1^ maleic acid, 420 μl L^−1^ lactic acid and 500 μl L^−1^ acetic acid) was added and the pH was adjusted to 1.3 with concentrated hydrochloric acid. After sonication in 37 °C ultrasound water-bath for 1 h, the supernatant (10 mL) was collected and stored in 4 °C for further analysis.

Prior to the intestinal digestion step, the pH of the gastric digests was raised to 7 by saturated sodium bicarbonate titration. 10 mL GI solution (0.4 g L^−1^ trypsin, 0.1 g L^−1^ amylase, and 1.5 g L^−1^ porcine bile) was added into the adjusted gastric digestive fluid. After a 4 h ultrasound treatment in a 37 °C water bath, the solution was placed in an ice water bath to stop the enzyme reaction. Finally, the gastrointestinal extracts were centrifuged at 10,000 rpm for 5 min and filtered through a 0.45 μm Millipore syringe filter. The extracts were stored at 4 °C for further analysis.

### 3.6. Determination of Total As

Total As from diverse sources was determined, including uncooked edible fungi, cooked edible fungi samples, CRMs, edible fungi soup, and stomach and gastrointestinal extracts. A total of 0.3 g sample or 3 mL digested supernatant was digested with 6 mL nitric acid (65%, wt%) and 1 mL hydrogen (30%, wt%) peroxide until complete transparency was achieved. The digestion was performed in a microwave digester with the following digestion procedure: 10 min at 120 °C; 10 min at 160 °C; 25 min at 190 °C. Afterwards, the digested solution was diluted to 20 mL with pure water and stored at 4 °C until further analysis.

Helium gas was used in the collision reaction cell to eliminate interference of other elements on As in the ICP-MS measurements. ^72^Ge (1000 μg L^−1^) was used as an internal standard for ICP-MS analysis of total As. The As content of each sample was quantified by an external calibration curve, which was repeated in triplicate.

### 3.7. As Speciation Analysis

HPLC-ICP-MS was used to determine the speciation of As in raw, soaked, stir-fried and boiled edible fungi and bioaccessible extracts. Ammonium carbonate was used as the mobile phase for gradient elution and six species of As could be well separated within 8 min. Operating conditions are listed in Table 4.

#### 3.7.1. Sample Preparation of Raw and Cooked Edible Fungi

Here in, 0.25 g of the samples with 20 mL ultrapure water was sonicated in a 60 °C water bath for 1 h. The mixture was cooled down to room temperature and centrifuged at 8000× *g* for 10 min. Prior to analysis of As speciation, the supernatant was filtered through a 0.45 μm Millipore syringe filter.

#### 3.7.2. As Speciation Analysis of Bioaccessible Extracts

As speciation in gastric and gastrointestinal extracts were determined by HPLC-ICP-MS. Operating conditions are listed in Table 1.

### 3.8. Statistical Analysis

The bioaccessibilities of As and As speciation were calculated as a percentage using the following equation:

BA (%) = [As in G or GI fraction]/[As in sample] × 100%(1)

where BA (%) is the bioaccessibility of As or bioaccessibility of As speciation; [As in G or GI fraction] is the As or certain As speciation concentration in the gastric or gastrointestinal fraction after *PBET* extraction;and [As in sample] is total As concentration after the microwave-assisted acid digestion procedure.

All data for the edible fungi samples were expressed as dry weight. All assays were performed at least in triplicate. Data were summarized as means and standard deviations (mean ± SD), and statistically analyzed using analysis of variance (ANOVA) at the 95% confidence level.

## 4. Conclusions

In this paper, the effects of different cooking methods on As and its speciation in two types of raw wild edible fungi (*Pleurotus citrinipileatus* and *Agaricus blazei Murill*) were investigated for the first time, and the bioaccessibility of As and its speciation in wild edible fungi was further investigated. It was found that As (63.5–98.1%) and As species (AsB. DMA, MMA, iAs) (81.3–151%) in wild edible fungi have high bioaccessibility, which supports a better assessment of the health risk of consuming wild edible fungi. To simulating people’s daily dietary habits, raw edible fungi were soaked before consumption and then processed by stir-frying and boiling cooking methods. Soaking of raw *Pleurotus citrinipileatus* and *Agaricus blazei Murill* released about 35.2–60.9% of the total As in water. The released As species in the water were the same as the initial As species in the edible fungi. Therefore, the risk of ingesting As in wild edible fungi can be reduced by soaking them during the daily consumption of raw wild edible fungi. In addition, the study found the As content of wild edible fungi decreases during cooking. Boiling can minimize the As content in edible fungi, which can release some As from wild edible fungi into the cooking water. Thereofre, when consuming wild edible fungi, the cooking method of boiling should be adopted, while reducing the intake of fungi soup.

At present, there are many kinds and quantities of wild edible fungi known, but only two kinds of wild edible fungi were selected for research in this study, which is not systematic and comprehensive enough, and can be further improved in subsequent research.

## Figures and Tables

**Table 1:** Accuracy and repeatability of the method for evaluating total As. Total As content is expressed as As μg kg^−1^ dry mass (mean ± SD, *n* = 6).

Certified Reference Material	Matrix	Accuracy		Recovery (%)	Repeatability (RSD %)
		Certified Value (μg kg^−1^)	Measured Value (μg kg^−1^)		
NIST SRM 1568b	Rice	285 ± 14	291.6 ± 4.7	102.7 ± 1.7	1.6
ERM-BC211	Rice	260 ± 13	256.2 ± 2.2	98.6 ± 0.8	0.8

**Table 2:** Concentration of total As and its speciation in raw, soaked, stir-fried, and boiled edible fungus and in boiling water, soaking solution. Concentrations expressed as As mg kg^−1^ dry mass (mean ± SD, *n* = 3).

Sample	Cooking Treatment	Total As	AsB	DMA	MMA	iAs
*Pleurotus* *citrinipileatus*	Raw	13.3 ± 0.6	0.078 ± 0.001	0.265 ± 0.02	0.273 ± 0.01	10.3 ± 0.4
	Soaked	6.45 ± 0.08	0.067 ± 0.003	0.105 ± 0.002	0.095 ± 0.002	5.28 ± 0.1
	Soaked + Stir-fried	5.93 ± 0.2	0.066 ± 0.002	0.121 ± 0.002	0.224 ± 0.007	4.57 ± 0.05
	Soaked + Boiled	2.74 ± 0.04	0.015 ± 0.0001	0.040 ± 0.001	0.054 ± 0.0001	1.92 ± 0.009
	Boiling water	6.47 ± 0.005	0.073 ± 0.001	0.179 ± 0.0004	0.185 ± 0.004	5.90 ± 0.03
	Soaking solution	4.68 ± 0.09	0.049 ± 0.002	0.220 ± 0.005	0.248 ± 0.005	3.82 ± 0.04
*Agaricus blazei Murill*	Raw	7.37 ± 0.02	5.48 ± 0.03	0.060 ± 0.001	0.411 ± 0.008	0.094 ± 0.004
	Soaked	3.00 ± 0.1	2.33 ± 0.01	0.040 ± 0.001	0.277 ± 0.002	0.064 ± 0.003
	Soaked + Stir-fried	2.00 ± 0.07	1.51 ± 0.03	0.036 ± 0.001	0.216 ± 0.008	0.060 ± 0.002
	Soaked + Boiled	0.49 ± 0.02	0.23 ± 0.003	0.008 ± 0.0002	0.040 ± 0.0005	0.011 ± 0.001
	Boiling water	2.04 ± 0.02	1.48 ± 0.01	0.034 ± 0.002	0.154 ± 0.005	0.073 ± 0.003
	Soaking solution	4.49 ± 0.1	3.39 ± 0.02	0.040 ± 0.001	0.196 ± 0.004	0.073 ± 0.003

**Table 3:** Bioaccessibility (BA%) of the total As and its speciation in G and GI fractions of treatment samples.

Sample	G and GI Fractions	Bioaccessibility (BA%)				
		Total As	AsB	DMA	MMA	iAs
*Pleurotus* *citrinipileatus*	Raw G	74.5 ± 1.1	99.0 ± 2.7	83.8 ± 6.1	79.9 ± 2.5	90.8 ± 1.5
	Raw GI	78.2 ± 1.0	99.3 ± 1.8	81.9 ± 5.6	81.3 ± 1.4	99.5 ± 1.4
	Soaked G	75.0 ± 1.4	84.3 ± 1.3	92.1 ± 3.8	82.0 ± 1.4	87.2 ± 1.6
	Soaked GI	83.5 ± 2.0	101.5 ± 1.0	100.8 ± 4.9	95.1 ± 2.3	97.0 ± 2.3
	Soaked + Stir-fried G	73.7 ± 0.3	84.5 ± 2.6	88.6 ± 3.3	79.1 ± 0.03	87.3 ± 0.4
	Soaked + Stir-fried GI	81.0 ± 0.9	95.0 ± 2.3	95.5 ± 1.5	98.1 ± 2.2	95.6 ± 1.4
	Soaked + Boiled G	64.4 ± 1.3	89.8 ± 1.7	89.2 ± 1.3	64.9 ± 2.1	86.6 ± 1.9
	Soaked + Boiled GI	70.8 ± 0.6	106.0 ± 2.3	100.8 ± 1.4	79.8 ± 2.5	94.6 ± 1.0
*Agaricus blazei Murill*	Raw G	93.0 ± 1.0	95.7 ± 1.1	88.3 ± 1.3	77.1 ± 1.1	96.1 ± 0.9
	Raw GI	98.1 ± 1.1	99.2 ± 1.2	97.8 ± 1.3	104.8 ± 3.1	102.7 ± 0.1
	Soaked G	79.0 ± 2.2	90.9 ± 2.8	82.4 ± 2.4	55.0 ± 2.2	109.8 ± 1.0
	Soaked GI	90.3 ± 2.0	97.3 ± 2.6	98.2 ± 2.3	114.5 ± 1.5	151.0 ± 0.5
	Soaked + Stir-fried G	80.6 ± 0.7	91.4 ± 0.6	81.5 ± 1.0	64.6 ± 1.5	100.8 ± 3.2
	Soaked + Stir-fried GI	92.0 ± 0.8	98.6 ± 0.7	97.2 ± 1.0	106.5 ± 1.9	139.2 ± 4.6
	Soaked + Boiled G	51.7 ± 0.4	96.2 ± 0.7	82.4 ± 2.9	46.0 ± 1.3	68.6 ± 3.2
	Soaked + Boiled GI	63.5 ± 1.1	105.1 ± 2.0	116.7 ± 2.7	111.4 ± 3.8	147.2 ± 2.9

**Table 4:** Instrumental parameters for ^75^As ICP-MS detection.

ICP-MS Parameters			
RF power (W)	1550		
Carrier gas flow (L min^−1^)	1.05		
Sampling depth (mm)	8.0		
He gas flow (mL min^−1^)	3.6		
Auxiliary flow (L min^−1^)	0.46		
Spray chamber temperature (°C)	2.0		
Peristaltic pump speed (rps)	0.30		
**HPLC Parameters**			
Analytical column	Dionex IonPac As7		
Sample injection volume (μL)	20		
Mobile phase flow (mL min^−1^)	1.2		
Mobile phase A	20 mM Ammonium carbonate		
Mobile phase B	100 mM Ammonium carbonate		
Gradient elution ramp:			
Time (min)	Flow rate (mL min^−1^)	A%	B%
0.000	1.2	100	0
1.599	1.2	100	0
1.600	1.2		100
5.599	1.2		100
5.600	1.2	100	
8.000	1.2	100	

## Data Availability

The data presented in this study are available on request from the corresponding author. The data are not publicly available due to privacy of study participants.

## Sample Availability

Samples of the compounds are not available from the authors.
